# The role of hospitalists in bedside ethics education for medical trainees

**DOI:** 10.1002/jhm.70337

**Published:** 2026-04-26

**Authors:** Holland Kaplan

**Affiliations:** ^1^ Center for Medical Ethics and Health Policy Baylor College of Medicine Houston Texas USA; ^2^ Section of General Internal Medicine Baylor College of Medicine Houston Texas USA; ^3^ Ben Taub Hospital Harris Health System Houston Texas USA

## Abstract

Hospitalists are well‐positioned to teach ethics to trainees at the bedside, traversing the chasm between preclinical instruction and real‐world patient care. This paper proposes a structured, generalizable approach to ethics education for hospitalists. Rooted in principles from the One‐Minute Preceptor approach, the *NAME* framework—*N*ame the ethical issue, *A*nchor the discussion in ethical standards, *M*ap relevant values and uncertainties, and *E*ngage trainees in supervised action and reflection—emphasizes practical ethical reasoning as an important part of clinical care. This framework is illustrated across common inpatient scenarios to show how hospitalists can explicitly teach ethics without disrupting established structures of rounds.

## INTRODUCTION

Since the emergence of hospital medicine in the 1990s, hospitalists have become central to medical education, particularly in inpatient settings where trainees spend significant portions of their clinical training.^1^ This position creates opportunities for ethics education that can connect trainees' theoretical knowledge acquired in preclinical courses with clinical practice. Because hospitalists regularly encounter common ethical dilemmas, their daily interactions with trainees provide numerous opportunities for teaching ethics in real‐time.^2^

Although clinical integration of ethics education has improved over time, traditionally, medical ethics education has relied heavily on classroom‐based instruction during preclinical years. Integrating ethics education into clinical practice settings has proven consistently challenging.^3^ Dr. Mark Siegler called for ethics education rooted in the Oslerian tradition of bedside rounding in 1978, arguing that the ethical dimensions of patient care cannot be approximated by case discussions and classroom‐based education alone. His statement reflects the real‐world uncertainty, moral distress, and relational dynamics with patients and families that cannot be approximated by case studies.^4^ The patient's bedside is, of course, where ethical questions most vividly arise and where teaching opportunities are often missed.^5^, ^6^, ^7^

There have been limited empirical studies evaluating the frequency and effectiveness of hospitalist‐led ethics education in the clinical setting, but there has been signal that trainees value bedside ethics education. In a cross‐sectional survey of residents who completed pediatrics or medicine/pediatrics residencies, respondents indicated that discussions with attending physicians supervising the care of their patients had the most impact on their ethics education in residency.^8^ Surveys have indicated that trainees value bedside, case‐based, and faculty role‐modeling approaches more than abstract preclinical instruction.^9^ Given that hospitalists inevitably model the ethical dimensions of clinical practice, this paper emphasizes making ethical reasoning explicit and reflective rather than relying on trainees' implicit observation via a structured approach—the *NAME* framework.

## FOUNDATIONS FOR BEDSIDE ETHICS TEACHING

Despite being well‐positioned to provide ethics education, most hospitalists have received minimal post‐training ethics education.^10^ Additionally, ethics topics are often perceived as less amenable to traditional teaching approaches or guideline‐based consensus than routine clinical topics.^11^ However, it is important to note that effective bedside ethics education does not require hospitalists to be trained philosophers or experts in moral theory. Although abundant systematic approaches to ethical reasoning have been developed,^12^, ^13^, ^14^, ^15^ few of these have been broadly integrated into bedside care. Much of practical clinical ethics involves applying established principles and professional guidelines to real‐world patient care rather than generating novel insights.

Importantly, hospitalists can also help trainees distinguish between situations addressed by clear ethical guidelines and more challenging scenarios that represent genuine ethical uncertainty. For the former, teaching may focus on reinforcing broadly accepted principles, such as respect for autonomy and beneficence. For the latter, hospitalists can demonstrate how to identify and analyze true ethical quandaries, cases where competing values or obligations may preclude a single “right” answer. In both instances, the hospitalist's role is not to resolve every ethical dilemma or to always “know the answer,” but to normalize ethical reflection and uncertainty as an important part of clinical and ethical reasoning and professional practice. Given that there is often uncertainty involved in identifying and managing ethical issues, bedside ethics education may be a particularly fruitful realm to prepare trainees for managing uncertainty, an ongoing challenge.^16^

## STRATEGIES FOR INTEGRATION WITH CLINICAL AND EDUCATIONAL WORKFLOWS

Rather than relying on scenario‐specific discussions, hospitalists can engage in bedside ethics teaching using a simple framework that can be generalized across clinical contexts. Drawing on established models of clinical teaching, such as the One‐Minute Preceptor (OMP), a simple framework for hospitalist‐led ethics education may be proposed: the *NAME* framework—*N*ame the ethical issue, *A*nchor the discussion in ethical standards, *M*ap relevant values and uncertainties, and *E*ngage trainees in supervision and reflection.

The *NAME* approach adapts core parts of the OMP to ethics education. When a hospitalist or trainee explicitly *N*ames an ethical issue, it helps the team identify the ethical nature of a scenario, which may otherwise have remained unnamed, and thus switch the team's approach to an ethical framework. This is similar to OMP's first step to “get a commitment,” because it engages the whole team in a particular framing of the issue. In the same way that OMP instructs “probing for supporting evidence,” encouraging trainees to *A*nchor the discussion in ethical standards (e.g., professional norms or guidelines, widely accepted ethical principles, legal requirements) enables them to provide “evidence” for a course of action even without extensive moral or philosophical expertise. Helping trainees *M*ap competing values between involved parties (e.g., patient's focus on longevity vs clinician's concern about beneficence and non‐maleficence) and uncertainties (e.g., unclear prognosis, patient's limited understanding of care plan) clarifies the areas of tension in the ethical dilemma. This mirror's the OMP strategy of “teaching general rules,” as it elucidates the structure of the ethical dilemma and models transferable analytical skills. Lastly, *E*ngaging trainees directly via supervised action and reflection (e.g., conducting a capacity assessment, leading a goals‐of‐care discussion) reinforces the practicality of ethical reasoning. In OMP, “reinforcing was what done well” and “correcting mistakes” similarly promotes deliberate practice. Table 1 frames several cases with ethical dimensions using the *NAME* framework for clinical teaching across multiple common inpatient scenarios.

**Table 1:** Using the NAME approach in bedside ethics education.

Clinical scenario	***N*** ame the ethical issue	*A*nchor the discussion in ethical standards	*M*ap relevant values and uncertainties	*E*ngage the trainee in supervision and reflection
A 58‐year‐old man with heart failure wants to leave against medical advice, expressing frustration with his care.	Decision‐making capacity	Elements of capacity, that is, choice, understanding, appreciation, reasoning	Explore patient values (e.g., autonomy vs. safety) and uncertainty related to illness	Trainee conducts supervised capacity assessment and debriefs with the team
A 72‐year‐old woman with schizophrenia and limited capacity intermittently refuses antibiotics; her daughter insists on continuing treatment.	Surrogate authority	Legal surrogate hierarchy and ethical standards (i.e., substituted judgment, best interests)	Discuss daughter's concerns and sources of decisional conflict, involve patient to the extent possible	Trainee discusses surrogate decision‐making role and limitations in treating over objection with the daughter and reflects with the team
The parents of a 5‐year‐old girl with leukemia want to pursue alternative treatments instead of chemotherapy.	Tension between parental authority and child's best interests	Best interest standard and thresholds for medical neglect	Explore parental values, fears, and uncertainty about prognosis and treatment	Trainee discusses negotiation strategies with team and is supervised in conversation with parents regarding treatment
An 82‐year‐old man with severe dementia and recurrent aspiration pneumonia is receiving PEG tube placement per his son's request	Informed consent and voluntariness	Requirements for valid informed consent and professional guidelines on PEG placement^17^	Clarify goals of care, prognostic uncertainty, and son's short‐term and long‐term expectations	Trainee conducts supervised goals of care conversation with subsequent debrief
A 67‐year‐old man with metastatic pancreatic cancer asks, “Am I dying?” His son requests that the team avoid discussing the prognosis with his father.	Disclosure and truth‐telling	Patient's right to information and standards for disclosure	Discuss rationale for patient's preferences, son's concerns, and emotional tensions	Trainee negotiates decision‐making paradigm with patient and son, and receives supervision and feedback in ongoing decision‐making
A 71‐year‐old woman with mild cognitive impairment and no surrogate is scheduled for surgery and will lack capacity post‐operatively.	Preventive ethics—Anticipating future incapacity	Legal surrogate hierarchy and advance directive options	Clarify patient's values and preferences as contingency planning prior to loss of capacity	Trainee assists patient in completing an advance directive
A 15‐year‐old girl admitted for pyelonephritis reports symptoms of a sexually transmitted infection and requests that the medical team not inform her parents.	Confidentiality	State law and professional guidelines on minor consent	Consider patient's priorities and practical constraints (e.g., medical record access by parents)	Trainee discusses disclosure strategies with team and is supervised in counseling the patient

Abbreviations: NAME, *N*ame the ethical issue, *A*nchor the discussion in ethical standards, *M*ap relevant values and uncertainties, and *E*ngage trainees in supervised action and reflection; PEG, percutaneous endoscopic gastrostomy.

Finally, as with other aspects of clinical education, bedside ethics teaching can be reinforced with curated resources that trainees can engage with independently. Major professional medical organizations publish guidelines on ethics topics commonly encountered by hospitalists, including pediatric shared decision making,^18^ potentially inappropriate treatment,^19^, ^20^ cardiopulmonary resuscitation,^21^ and provision of artificial nutrition and hydration.^17^ State and federal laws are also often applicable to ethical challenges involving surrogate decision making, non‐beneficial treatment, and code status discussions. These guidelines may also be used when *A*nchoring discussions in ethical standards in the *NAME* approach. Hospitalists frequently augment routine clinical teaching with materials such as podcasts, academic literature, and clinical guidelines, all of which also exist for common ethical issues. Podcasts such as *Core IM* and *Bioethics for the People* address frequently encountered dilemmas, including decision‐making capacity assessment and treatment over objection.

## CHALLENGES AND BARRIERS

Hospitalists often face competing demands between patient care, documentation requirements, meeting quality metrics, and educational responsibilities. Clinical productivity pressures and intense patient care responsibilities limit hospitalists' time available for ethics teaching.^22^ Broader institutional factors can also impede ethics education, such as lack of protected time for teaching, insufficient support for faculty development, limited collaboration with clinical ethicists, and inadequate assessment tools for ethical competencies. While these challenges pervade clinical teaching broadly, ethics may be particularly vulnerable due to less standardized curricula and lower institutional prioritization. Some of these barriers may be alleviated by integrating ethics teaching into existing rounding and teaching structures via the *NAME* framework, providing hospitalists with structured education in ethical reasoning (e.g., faculty development sessions, grand rounds), and establishing formal collaboration between hospitalists and clinical ethicists.

Importantly, the *NAME* framework is not intended to be applied to every patient encounter or favored over other educational priorities on rounds. It is intended to be used when a hospitalist recognizes that there are important ethical dimensions of care that must be addressed to provide good patient care. While the *NAME* framework has similarities to effective general clinical teaching strategies, its framing, focused on ethical issues, makes it distinct. Clinical reasoning frameworks focus on developing the best approach for clinical dilemmas, whereas the *NAME* framework centers on questions involving ethical issues. The *NAME* framework can cue hospitalists to both recognize and educate trainees on ethical issues that arise in everyday clinical care that might otherwise remain unstated.

## CONCLUSION

As Dr. Eric Cassell wrote in 1973, “To conceive of a physician practicing his profession without constant ethical decision‐making is to conceive of a physician operating in a cultural vacuum and caring for a collection of static facts wrapped in human form. Medicine is inherently moral.”^23^ The ethical dimensions of patient care cannot be separated from clinical practice. Hospitalists are a significant and underutilized resource for ethics education in clinical practice for medical trainees. Their proximity to learners, exposure to ethical dilemmas, and commitment to education position them to provide meaningful ethics instruction in real‐time, which can be integrated into existing rounding and teaching paradigms via the *NAME* framework outlined in this paper. However, realizing this potential also requires addressing important barriers, including faculty preparation, time constraints, and institutional support. By addressing these needs, hospitalists can serve as central contributors to the ethical development of trainees.

## CONFLICT OF INTEREST STATEMENT

The author declares no conflicts of interest.

## Data Availability

Data sharing is not applicable to this article as no datasets were generated or analyzed during the current study.
